# Access and barriers to safe anesthesia in primary health care settings in India

**DOI:** 10.6026/973206300220311

**Published:** 2026-01-31

**Authors:** Manish Kumar Dwivedi, Divas Sinha, Pramod Kumar Verma

**Affiliations:** 1 Department of Anaesthesiology, GMC Singrauli, Singrauli, Madhya Pradesh, India; 2 Department of Anaesthesiology, Ram Krishna Medical College and Research Centre, Bhopal, Madhya Pradesh, India; 3 Department of Community Medicine, Chhindwara Institute of Medical Sciences, Chhindwara, Madhya Pradesh, India

**Keywords:** Anesthesia services, primary health care, barriers, access to care, patient safety, rural health, India

## Abstract

The availability of safe anesthesia services is an essential part of necessary surgical care, but it is a pressing issue that has been poorly considered in the primary health care environment in low and middle-income countries. Therefore, it is of interest to assess the availability of safe anesthesia services, their accessibility, and obstacles of these services in primary health care in three Indian states. Between January 2022 and August 2022, 156 primary health centres (PHCs) and 312 anesthesia providers were surveyed by using structured questionnaires and facility assessment tools. Findings showed that not all PHCs had operational anesthesia processes (38.5 percent), and not all PHCs had trained anesthesia staff (62.8 percent). The major obstacles were identified as the lack of proper infrastructure (71.2%), lack of skilled labour (68.6%), unavailability of necessary drugs (54.5%), and lack of standard procedures (47.4%). Rural amenities had much worse access than urban centres (p<0.001).

## Background:

The right to safe anesthesia and surgical services is a key element of universal health services but an approximated five billion individuals worldwide have no access to safe, cheap, and in-time surgical operation [1]. Lancet Commission on Global Surgery highlighted the need to build the anesthesia capacity at the primary and secondary care levels as a key to solving the global burden of surgical diseases [2]. In India where almost 68 percent of the population lives in rural houses, the gap between anesthesia service provision in rural and urban environments poses a significant challenge in terms of the public health [3]. In India, the medical problems of surgical morbidity contribute to about 15 percent of the total years of disability-adjusted life (DALY), with a large percentage of them needing anesthesia services [4]. In the three level health care systems in India, the first referral unit is the primary health centres (PHCs), and in theory, they are capable of performing simple surgical procedures such as emergency obstetric care, trauma management and minor surgical procedures [5]. Nevertheless, the real delivery of anesthesia services at this stage is appallingly unsatisfactory which adds to delayed care, avoidable death and disastrous health spending [6]. Recent reports have reported major gaps in workforce in the field of anesthesia in the world with the worst hits in South Asian nations [7]. The population density of anaesthesiologists in India is estimated at 1.3 per 100,000 inhabitants which is a very low number considering the minimum number of 5 per 100,000 recommended [8]. In addition to the workforce issues, infrastructure, and equipment are not available, insufficient training, and structural issues exacerbate the issue of anesthesia service delivery in resource-constrained environments [9]. Past studies in sub-Saharan Africa and Southeast Asia have found several obstacles to safe anesthesia such as a shortage of monitoring facilities, difficulties in provision of oxygen, shortage of drugs, and absence of uniform safety guidelines [10, 11]. Nevertheless, there are limited studies that specifically investigate the condition of anesthesia services in Indian primary health care facilities. The majority of studies done have concentrated on tertiary care facilities or particular process aspects as opposed to analyzing the access and obstacles at the primary care level in a systematic way [12]. The knowledge of the present situation within the anesthesia services and the identification of the distinct barriers will be necessary to formulate tailored interventions to enhance surgical care provision at the grassroots level. This becomes especially important when we are talking about the National Health Mission of India and the newly introduced Ayushman Bharat program, the goal of which is to empower the primary health care infrastructure [13]. Therefore, it is of interest to systematically analyze the existing level of access to safe anesthesia services in primary health care institutions in three states in India and to define the main obstacles to service delivery in order to inform policy and programmatic interventions.

## Materials and Methods:

## Study design and setting:

This cross-sectional observational study was conducted from January 2022 to August 2022 across primary health centres in three states representing different geographic and developmental contexts in India: Maharashtra (western region), Uttar Pradesh (northern region), and Karnataka (southern region). The study employed a multi-stage stratified random sampling approach to ensure geographic and demographic representation.

## Sample size calculation:

Sample size was calculated using the formula for cross-sectional studies with expected proportion of facilities with adequate anesthesia services at 35% (based on pilot data), 95% confidence interval, and 5% margin of error. The calculated minimum sample size was 350 facilities. Accounting for 10% non-response rate, the target sample was 385 facilities. Ultimately, 156 PHCs were surveyed along with 312 anesthesia providers (including anaesthesiologists, nurse anaesthetists, and medical officers performing anesthesia).

## Sampling strategy:

From each state, districts were randomly selected using probability proportional to size sampling. Within each district, PHCs were stratified into rural and urban categories, and random selection was performed using computer-generated random numbers. A total of 52 PHCs were selected from each state.

## Inclusion and Exclusion Criteria:

## Inclusion criteria:

(1) Government-operated primary health centres functioning for at least two years; (2) Facilities designated to provide basic surgical services; (3) Health care providers involved in anesthesia administration or perioperative care with at least six months of experience.

## Exclusion criteria:

(1) Private facilities; (2) PHCs non-functional during the study period; (3) Facilities exclusively providing outpatient services without any surgical capability; (4) Providers who declined consent or were unavailable after three contact attempts.

## Data collection tools:

Data collection utilized three standardized instruments: (1) WHO-AMDISS (Anesthesia Monitoring and Safety Delivery System) adapted facility assessment checklist examining infrastructure, equipment, drug availability, and safety protocols; (2) Structured provider questionnaire assessing training, experience, perceived barriers, and practice patterns; (3) Document review protocol for examining existing records, protocols, and adverse event reporting systems.

## Study variables:

## Primary outcomes:

Availability of functional anesthesia equipment, presence of trained anesthesia personnel, availability of essential anaesthetic drugs and existence of standardized safety protocols.

## Secondary outcomes:

Types of anesthesia services provided, adverse event rates, referral patterns, and provider-perceived barriers.

## Independent variables:

Facility location (rural/urban), bed capacity, annual patient load, staffing patterns, budget allocation, distance from secondary care facility, state-level characteristics, and administrative support.

## Data collection procedure:

Trained research assistants with medical backgrounds conducted facility assessments and provider interviews following standardized protocols. Facility infrastructure and equipment were directly observed and documented using structured checklists. Drug availability was verified through pharmacy stock registers. Providers were interviewed privately using structured questionnaires. Each facility visit lasted approximately 4-6 hours. Data quality was ensured through daily review, field supervision, and 10% random re-verification.

## Ethical considerations:

Ethical approval was obtained from the Institutional Ethics Committee (IEC/2021/PHC/456). Written informed consent was obtained from all participating providers. Facility heads provided institutional consent. Confidentiality and anonymity were maintained throughout data collection and analysis.

## Statistical analysis:

Data were entered into EpiData version 3.1 and analyzed using SPSS version 26.0. Descriptive statistics included frequencies, percentages, means, and standard deviations. Chi-square tests assessed associations between categorical variables. Independent t-tests and ANOVA compared continuous variables across groups. Binary logistic regression identified predictors of adequate anesthesia service availability, with adjusted odds ratios (AOR) and 95% confidence intervals reported. Statistical significance was set at p<0.05 (two- tailed).

## Results:

Of the 156 PHCs surveyed, 104 (66.7%) were located in rural areas and 52 (33.3%) in urban settings. The mean bed capacity was 18.4 ± 6.2 beds (range: 10-30). Mean annual surgical case load was 142.6 ± 89.3 procedures. Among 312 providers surveyed 28 (9.0%) were qualified anaesthesiologists, 76 (24.4%) were nurse anaesthetists and 208 (66.6%) were medical officers performing anesthesia as additional duty. The mean professional experience in anesthesia provision was 6.8 ± 4.2 years. Only 60 PHCs (38.5%) had functional anesthesia equipment meeting minimum standards. Essential monitoring equipment availability varied significantly: pulse oximeters were available in 89 facilities (57.1%), blood pressure monitors in 124 (79.5%), electrocardiogram machines in 52 (33.3%), and capnography in only 12 (7.7%). Functional oxygen supply systems were present in 94 facilities (60.3%). Dedicated operation theatres with appropriate infrastructure existed in 71 PHCs (45.5%). Table 1 presents the comprehensive infrastructure and equipment availability across surveyed facilities, stratified by location. Only 58 PHCs (37.2%) had dedicated trained personnel for anesthesia administration. The majority (62.8%) relied on medical officers with minimal or no formal anesthesia training. Among the 312 providers surveyed, only 142 (45.5%) had received formal training in anesthesia (duration ≥3 months). Continuous medical education (CME) participation in the past year was reported by 98 providers (31.4%). Training in advanced airway management was completed by 89 providers (28.5%), while basic life support certification was held by 156 (50.0%). Essential anaesthetic drug availability was suboptimal across facilities. Ketamine was available in 118 facilities (75.6%), while propofol was available in only 34 (21.8%). Local anaesthetics (lidocaine/bupivacaine) were available in 132 facilities (84.6%). Neuromuscular blocking agents were available in 48 facilities (30.8%). Reversal agents were stocked in 42 facilities (26.9%). Epinephrine and atropine were available in 145 (92.9%) and 138 (88.5%) facilities respectively (Table 2). Multiple barriers were identified through provider surveys and facility assessments. Infrastructure inadequacy was cited by 111 PHCs (71.2%), workforce shortage by 107 (68.6%), limited drug availability by 85 (54.5%), absence of standardized protocols by 74 (47.4%), inadequate training opportunities by 103 (66.0%), insufficient administrative support by 92 (59.0%), and lack of functional referral systems by 88 (56.4%). Financial constraints and budget limitations were identified in 96 facilities (61.5%) (Table 3). Among facilities with anesthesia capability, spinal anesthesia was the most commonly provided technique (78.2%) followed by local anesthesia (94.2%), ketamine- based general anesthesia (46.8%), and regional blocks (23.1%). Only 15.4% of facilities reported providing balanced general anesthesia with endotracheal intubation. Emergency caesarean sections, fracture management, and minor surgical procedures constituted the primary surgical case mix. The mean referral rate for surgical cases requiring anesthesia was 64.3 ± 18.7%.

## Discussion:

This multi-state assessment shows that there is a great lack of access to safe anesthesia services in the primary health care stage in India, and there are extreme rural-urban inequalities. PHCs had 38.5 percent functional anesthesia and only 37.2 percent trained personnel, which demonstrated severe deficiencies in infrastructure and human resources. The results are consistent with the world problem of the surgical disease literature that documents a considerable proportion of preventable deaths and disability due to a lack of accessibility to surgical and anesthesia services in primary care and health facilities [14]. Insufficiency of qualified anesthesia workforce as seen in this study (62.8 percent of the facilities have no specialized staff) is supported by prior evaluations of workforce in low- and middle-income nations. Similar shortages in the workforce were recorded in a systematic review by Karydi *et al.* in both African and Asian countries, with the addition that task-shifting and training of non-physician anesthesia providers is one of the possible options to increase access [15]. The fact that 66.6 percent of the anesthesia administration is carried out by medical officers who have the least formal training has great implications on patient safety and the need to establish structured training and competency-based certification systems is under urgent consideration. The most common barrier (71.2 percent) was infrastructure inadequacy, which is aligned with the results of the reports of the National Health Systems Resource Centre on the PHC functionality in India [16]. Cookson *et al.* demonstrates inverse care law persists in LMICs including India, where neediest rural populations receive least services, creating inequity cycles in health access [17]. Availability of essential monitoring equipment was also of particular concern as capnography was available in only 7.7% of facilities. WH and World Federation of Societies of Anaesthesiologists have cited pulse oximetry and blood pressure monitoring to be minimum standards of safe anesthesia [18]. The fact that we have found that 42.9% of the facilities do not have pulse oximeters means that this is a serious safety shortfall because pulse oximetry has been shown to decrease mortality caused by anesthesia up to 70% in scarce resources facilities.

The availability of drugs, especially to critical anaesthetics such as propofol (21.8%), neuromuscular blockers (30.8%), and similar, is a limiting factor which significantly limits the list of safe anesthesia procedures which could be offered. The number of stock-outs (60.3% in some cases) is high, which is evidence of the system failure in the supply chain that should be addressed urgently. These results are in line with the results published by Kassebaum and colleagues in their international evaluation of surgical infrastructure and consumables [2]. The logistic regression model has determined a number of changeable predictors of sufficient availability of anesthesia services. Dedicated anesthesia personnel, service adequacy (AOR=4.21, p<0.001) is of high significance, which highlights the paramount importance of workforce development. Likewise, the fact that it is positively related to regular training programs (AOR=3.15, p=0.002) implies that regular continuing education programs might significantly enhance service capacity. Another important predictor that was identified was administrative support (AOR=2.68, p=0.005), whereby governance and management have a role to play in facilitating service delivery. The overwhelming use of spinal anesthesia (78.2%), as well as ketamine based methods (46.8%), is a logical way of adapting to the limited resources, as the two methods do not need complex equipment and monitoring. Nevertheless, the nature of the present availability of full general anesthesia facilities (15.4) limits the type of surgical case that can be complex and the cases referral has to be made, which may delay the provision of care and increase the costs imposed on patients. There are a number of limitations in this research. Causal inference is excluded by the fact that the cross-sectional design is cross-sectional. Evaluation was partly based on self-reporting by the providers, which might have caused social desirability bias. Patient outcomes and patient satisfaction were not directly measured in the study. Only three states were chosen, and they are geographically varied; thus, it is possible that such a sampling will not be representative of all Indian states. The narrowness of the government facilities is exclusive of the private sector that offers a significant health care in India. The results of this study can be applied in a number of ways to policy. To begin with, there is a need to invest in PHCs infrastructure, especially in rural locations. Second, the systematic development of the workforce based on the models of task-shifting and systematic training of the non-physician providers should be prioritized. Third, it is important to reinforce the pharmaceutical supply chains to guarantee the steady supply of necessary anesthetics. Fourth, standardized safety procedures and surveillance systems must be enforced and promoted. Lastly, there is the need to have improved administration and accountability systems to facilitate the translation of resource allocation into functional service provision.

## Conclusion:

There are significant gaps with regards to the accessibility of safe anesthesia services in primary health care facilities in India. Special anesthesia staff, sufficient budgetary allocation, frequent training sessions, and administration support proved to be serious predictors of service sufficiency. Such results highlight the necessity of multi-pronged measures such as improving infrastructure, workforce management by task-shifting and training, strengthening their supply chain, standardizing protocols, and reforming their governance.

## Figures and Tables

**Table 1:** Infrastructure and equipment availability in primary health centers (N=156)

**Infrastructure/Equipment Component**	**Total n (%)**	**Rural (n=104) n (%)**	**Urban (n=52) n (%)**	**p-value**
Functional operation Theatre	71 (45.5)	38 (36.5)	33 (63.5)	0.001
Anesthesia machine (functional)	60 (38.5)	30 (28.8)	30 (57.7)	<0.001
Oxygen supply system	94 (60.3)	55 (52.9)	39 (75.0)	0.008
Pulse oximeter	89 (57.1)	52 (50.0)	37 (71.2)	0.012
Blood pressure monitor	124 (79.5)	79 (76.0)	45 (86.5)	0.124
ECG machine	52 (33.3)	26 (25.0)	26 (50.0)	0.002
Capnography	12 (7.7)	4 (3.8)	8 (15.4)	0.01
Emergency drugs kit (complete)	78 (50.0)	44 (42.3)	34 (65.4)	0.007
Resuscitation equipment	82 (52.6)	48 (46.2)	34 (65.4)	0.024
Backup power supply	98 (62.8)	58 (55.8)	40 (76.9)	0.01

**Table 2:** Essential anaesthetic drug availability and barriers identified (N=156)

**Essential Anaesthetic Drugs**	**Available n (%)**	**Stock-outs in past 6 months n (%)**
Ketamine	118 (75.6)	67 (42.9)
Propofol	34 (21.8)	89 (57.1)
Thiopentone	28 (17.9)	94 (60.3)
Local anaesthetics	132 (84.6)	45 (28.8)
Neuromuscular blockers	48 (30.8)	78 (50.0)
Reversal agents	42 (26.9)	82 (52.6)
Opioid analgesics	56 (35.9)	71 (45.5)
Epinephrine	145 (92.9)	24 (15.4)
Atropine	138 (88.5)	32 (20.5)

**Table 3:** Provider-perceived barriers and predictors of adequate anesthesia services

**Barriers/Predictors**	**Frequency n (%)**	**Adjusted Odds Ratio (95% CI)**	**p-value**
**Provider-perceived barriers**			
Inadequate infrastructure	111 (71.2)	-	-
Workforce shortage	107 (68.6)	-	-
Limited drug availability	85 (54.5)	-	-
Lack of training opportunities	103 (66.0)	-	-
Insufficient administrative support	92 (59.0)	-	-
Absence of protocols	74 (47.4)	-	-
Poor referral systems	88 (56.4)	-	-
**Predictors of adequate services**			
Urban location	33/52 (63.5%)	2.84 (1.42-5.68)	0.003
Dedicated anesthesia personnel	58 (37.2%)	4.21 (2.08-8.53)	<0.001
Annual budget >5 lakhs INR	62 (39.7%)	2.97 (1.51-5.84)	0.002
Regular training programs	48 (30.8%)	3.15 (1.54-6.44)	0.002
Administrative support present	64 (41.0%)	2.68 (1.35-5.32)	0.005

## References

[1] Meara JG (2016). Int J Obstet Anesth..

[2] Nepogodiev D (2025). Lancet Health Policy..

[3] Ugargol AP (2023). Lancet Reg Health Southeast Asia..

[4] Shrime MG (2015). Lancet Glob Health..

[5] Bhatia MB (2021). Ann Glob Health..

[6] Menon N (2024). BMJ Glob Health..

[7] Kempthorne P (2017). Anesth Analg..

[8] Law TJ (2019). Indian J Anaesth..

[9] Dohlman LE (2020). Local Reg Anesth..

[10] Hadler RA (2016). World J Surg..

[11] Shahbaz S, Howard N (2024). PLOS Glob Public Health..

[12] Vijayaraghavan BKT (2022). PLoS One..

[13] https://nhm.gov.in/images/pdf/guidelines/iphs/iphs-revised-guidlines-2022/03_PHC_IPHS_Guidelines-2022.pdf.

[14] Alkire BC (2015). Lancet Glob Health..

[15] Karydi KI (2024). BMJ Open..

[16] Rao KD (2014). Health Policy Plan..

[17] Cookson R (2021). Lancet..

[18] Gelb AW (2018). Anesth Analg..

